# Next generation sequencing analysis of nine *Corynebacterium ulcerans* isolates reveals zoonotic transmission and a novel putative diphtheria toxin-encoding pathogenicity island

**DOI:** 10.1186/s13073-014-0113-3

**Published:** 2014-11-28

**Authors:** Dominik M Meinel, Gabriele Margos, Regina Konrad, Stefan Krebs, Helmut Blum, Andreas Sing

**Affiliations:** LGL, Bavarian Health and Food Safety Authority, Oberschleißheim, 85764 Germany; National Consiliary Laboratory on Diphtheria, Oberschleißheim, 85764 Germany; Laboratory for Functional Genome Analysis, Gene Center, Ludwig-Maximilians-University Munich, Munich, 81377 Germany

## Abstract

**Background:**

Toxigenic *Corynebacterium ulcerans* can cause a diphtheria-like illness in humans and have been found in domestic animals, which were suspected to serve as reservoirs for a zoonotic transmission. Additionally, toxigenic *C. ulcerans* were reported to take over the leading role in causing diphtheria in the last years in many industrialized countries.

**Methods:**

To gain deeper insights into the *tox* gene locus and to understand the transmission pathway in detail, we analyzed nine isolates derived from human patients and their domestic animals applying next generation sequencing and comparative genomics.

**Results:**

We provide molecular evidence for zoonotic transmission of *C. ulcerans* in four cases and demonstrate the superior resolution of next generation sequencing compared to multi-locus sequence typing for epidemiologic research. Additionally, we provide evidence that the virulence of *C. ulcerans* can change rapidly by acquisition of novel virulence genes. This mechanism is exemplified by an isolate which acquired a prophage not present in the corresponding isolate from the domestic animal. This prophage contains a putative novel virulence factor, which shares high identity with the RhuM virulence factor from *Salmonella enterica* but which is unknown in Corynebacteria so far. Furthermore, we identified a putative pathogenicity island for *C. ulcerans* bearing a diphtheria toxin gene.

**Conclusion:**

The novel putative diphtheria toxin pathogenicity island could provide a new and alternative pathway for Corynebacteria to acquire a functional diphtheria toxin-encoding gene by horizontal gene transfer, distinct from the previously well characterized phage infection model. The novel transmission pathway might explain the unexpectedly high number of toxigenic *C. ulcerans.*

**Electronic supplementary material:**

The online version of this article (doi:10.1186/s13073-014-0113-3) contains supplementary material, which is available to authorized users.

## Background

Diphtheria is the most severe disease attributed to coryneform bacteria [1]. Although *Corynebacterium diphtheriae* is the classical pathogen described to cause diphtheria, *Corynebacterium ulcerans* has also been found to cause diphtheria-like illness in humans. Moreover, in recent years cases of human diphtheria caused by *C. ulcerans* seem to outnumber those caused by *C. diphtheriae* in many industrialized countries, including the United Kingdom [2], France [3], the US [4] and Germany [5]. In contrast to *C. diphtheriae*, which to date has been found nearly exclusively in humans, *C. ulcerans* is often found in domestic animals, which are suspected to serve as reservoirs for possible zoonotic infection. Among those animals were cats, dogs and pigs [6-11]. Additionally, *C. ulcerans* has also been found in other non-domestic animals, such as cynomolgus macaques [12] and ferrets [13], and in game animals, such as wild boars and roe deer [14]. Although *C. ulcerans* is considered to be a zoonotic pathogen, molecular indication for zoonotic transmission has been found only in four instances, two of them involving dogs [9,15], one a cat [6] and one a pig [10].

Diphtheria is caused by diphtheria toxin (DT)-producing strains of the three *Corynebacterium* species, *C. diphtheriae*, *C. ulcerans* and *C. pseudotuberculosis.* DT is responsible for both the local form of diphtheria, which is characterized by a greyish pseudomembrane at the infection site both in respiratory or cutaneous disease, as well as the systemic symptoms, for example, neurological or cardiac manifestations. DT is a very potent toxin that is able to act on many different types of cells (reviewed in [16]). This Y-shaped protein toxin was shown by X-ray crystallography to consist of three domains [17]. The carboxy-terminal domain of the toxin serves as a receptor, which interacts with the heparin-binding epidermal growth factor precursor on the cell surface [18,19] and is therefore necessary for efficient endocytosis of DT into the cell. The translocator domain forms the middle part of the toxin and is able to integrate into the endosomal membrane upon the pH change after endocytosis, thereby transferring the amino-terminal, catalytically active part of the toxin into the cytoplasm. The active amino-terminal domain catalyzes the ADP-ribosylation of the translation factor EF-2 with the consumption of NAD and thereby irreversibly inhibits protein synthesis in the cell [20-22]. Remarkably, even a single DT molecule is sufficient to kill a eukaryotic cell [23].

However, not all isolates of *C. diphtheriae* and *C. ulcerans* are toxigenic. It has been reported that infection with a toxigenic phage can cause conversion by integration into the bacterial genome. Noteworthy, the DT encoding *tox* gene is located at the outer border of the integrated, linearized prophage genome. It is thought that the *tox* gene was acquired by the phage and might be transferred also to other phages [24]. The expression of the *tox* gene is controlled by the diphtheria toxin repressor (DtxR), which represses its transcription under high or normal Fe^2+^ concentrations [25]. DtxR is not encoded by the toxigenic phage, but on the bacterial chromosome [26]. Additionally, DtxR controls not only the toxin gene but also other genes for corynebacterial siderophores, heme oxygenase, and several other proteins [16]. The Fe^2+^ concentration is usually extremely low in the body fluids of humans or animals and DT is therefore produced by toxigenic strains [16].

Since we and others have registered over recent years many cases of toxigenic *C. ulcerans* causing diphtheria-like disease in humans, we aimed to analyze the toxigenic conversion of *C. ulcerans*. Resequencing data from nine *C. ulcerans* strains which were isolated from four human patients and their domestic animals showed that the bacteria strains were transmitted zoonotically. Moreover, we found that the pathogenic potential of *C. ulcerans* can change very rapidly due to infection by a phage containing a novel virulence gene, which was firstly described in *Salmonella*, and we also describe a novel DT-encoding putative pathogenicity island (PAI) which differs completely from the so far known toxigenic prophages of Corynebacteria.

## Methods

### Culture of bacteria and DNA isolation

*C. ulcerans* isolates were grown in liquid culture using Thioglycolat-Bouillon (37°C aerobic conditions). The *C. ulcerans* isolates were taken from the German Consiliary Laboratory on Diphtheria (NCLoD) isolate collection. The investigations were performed as part of public health outbreak investigations. Therefore, additional ethical approval was not required. Isolate species were determined by matrix-assisted laser desorption/ionization (MALDI)-time of flight (TOF) mass spectrometry and/or biochemical testing and the isolates were tested for toxigenicity by DT-PCR as described in [27]. The Elek test for DT expression was performed according to [28]. For next generation sequencing (NGS), 20 ml *C. ulcerans* culture was harvested by centrifugation and DNA was extracted after lysozyme digestion at 37°C for 15 minutes using a Maxwell 16 DNA extraction device (Promega, Mannheim, Germany). Bacteria were treated with lysis buffer containing Proteinase K and RNase for 2 h at 65°C and DNA purification was performed as described by the manufacturer.

### Genome sequencing, draft assembly and analysis

After quality control of the DNA, a tagmentation library was generated as described by the manufacturer (NexteraXT kit, Illumina, San Diego, CA, USA). The genomes were sequenced as multiplexed samples using a 2 × 250 bp V2 reaction kit on an Illumina MiSeq instrument reaching an average coverage of approximately 50-fold for all isolates. After quality control of the raw data, the reads were adapter clipped and quality trimmed and downstream analysis was carried out using a local instance of Galaxy [29-31]. We used SOAP denovo (v.1.0.0) for assembly of the genome [32] and BWA for Illumina (v.1.2.3) [33] for mapping the reads to the reference genome *C. ulcerans* 809 [34]. The mapping was refined using SRMA (v.0.2.5) [35]. SNPs were determined for the sequenced isolates and the published *C. ulcerans* genomes using VarScan (v.2.3.2) [36] and R (v.3.0.3, CRAN) [37]. The used R scripts are available upon request. Since we employed the *C. ulcerans* 809 genome as a reference, which carries a prophage in its genome, we excluded the region harboring the prophage from the analysis [34].

As we aimed to compare our resequencing data with the published finished genomes without losing quality information in our resequencing data, we only used SNPs which could be unambiguously identified in our sequenced dataset. This implies that the regions not covered by our re-sequencing are not included in the analysis. To prevent acceptance of false negative SNPs, we firstly determined a set of SNPs that could be called with very high quality (minimum coverage of 20 reads and at least 90% variant frequency) in at least one of our samples and compiled a list of trustworthy SNP positions in our sequenced genomes. In the next step, we used this list to determine if these SNPs are also present in the other isolates - that is, we analyzed all those positions of the trustworthy SNPs in all isolates by allowing identification of the presence of SNPs at the given position with lower quality criteria. The lower quality criteria were minimum coverage of two-fold with at least a variant frequency of >50%.

The first step ensures that we only consider positions within the genomes with reliable SNPs. The second step ensures that, upon identification of a SNP at a certain position in one isolate, the remaining isolates are not false negatives due to too little coverage - that is, the quality of SNP calling - at the corresponding position.

For the detailed analysis of matched isolates (isolates within a pair), we manually curated the intra-pair SNPs; that is, we excluded from both isolates SNPs that we were not able to correctly determine in one of the two strains due to missing data at the corresponding genomic position. Therefore, we deleted a SNP from the manually corrected list of an isolate if it was not possible to determine in the matched isolate whether there is a SNP or not at the corresponding position. Thereby we avoided false negative SNPs (that is, negative detection due to missing data), which would lead to possibly spurious differences between two isolates when comparing them. We did not perform manual curation for the inter-pair SNPs, since random inspection showed that only a very minor fraction of the SNPs in this category was due to coverage problems (less than 3 out of 1,000 SNPs). This is most likely caused by the fact that the critical positions where only one of the isolates has sufficient sequencing coverage are very small compared with the remaining genome and form an approximately constant false negative SNP background level, which only reaches a considerable fraction for a small number of real SNPs. For calculation of the phylogenetic trees, we exported the SNPs, and concatenated and constructed the phylogeny (neighbor joining) using MEGA 6.0 [38]. BRIG [39], Artemis [40] and IGV [41] were used for visualization of the data. Multi-locus sequence typing (MLST) SNP data for *atpA*, *dnaE*, *dnaK*, *fusA*, *leuA*, *odhA* and *rpoB* were extracted from the NGS dataset.

xBase was used for the annotation of the draft genome [42]. Contigs were sorted using Mauve [43] and concatenated using the genomic sequence of *C. ulcerans* 809 [34] as reference. xBase uses Glimmer for gene prediction [44], and tRNAScan-SE [45] and RNAmmer [46] for prediction of tRNAs and rRNAs. BLAST was used for annotation of the predicted proteins [47]. Prophages were searched using PHAST [48]. Therefore, we sorted our *de novo* assembled contigs and the contigs of FRC58 [49] versus the reference genome of *C. ulcerans* 809 and analyzed the concatenated sequences with PHAST. Annotated proteins were further analyzed with BLAST, HHPred [50] and InterPro [51] Multiple alignments were calculated with Clustal Omega [52] and visualized with Jalview [53].

### Next generation sequencing data

All sequencing data are available from the Sequence Read Archive [54] under experiment accession number SRX740276. The annotated region of the putative PAI is available at GenBank (KP019622).

## Results

### Toxigenic *C. ulcerans* outnumber toxigenic *C. diphtheriae*

**Figure 1:**
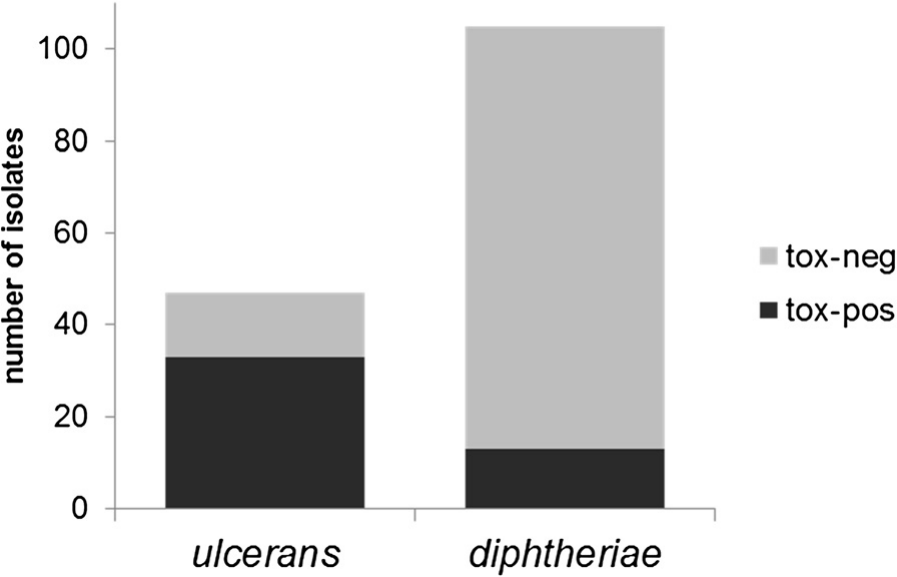
**Toxigenic and non-toxigenic** ***C. ulcerans*** **and** ***C.*** ***diphtheriae*** **isolates from 2011 to 2013.** *Corynebacterium* isolates sent to the NCLoD. Species and toxigenicity of the isolates were determined using MALDI mass spectroscopy and PCR, respectively. The isolates are derived from human patients and animals.

Wagner *et al*. [2] found that toxigenic *C. ulcerans* infections outnumber toxigenic *C. diphtheriae* infections in diphtheria patients in the United Kingdom. We wondered whether this phenomenon could be due to a higher proportion of toxigenic versus non-toxigenic *C. ulcerans* compared with the proportion of toxigenic versus non-toxigenic *C. diphtheriae.* Therefore, we analyzed the database of the NCLoD at the Bavarian Health and Food Safety Authority. The isolates analyzed here were sent for differentiation to the NCLoD by several clinical microbiology laboratories and as a caveat might not be representative of the whole *Corynebacterium* population in Germany and several of the Corynebacteria were isolated from animals. Among the 103 *C. diphtheriae* isolates sent to the NCLoD between 2010 and 2013, 13 (12.4%) were *tox*-positive (Figure 1). In contrast, a much higher proportion of *C. ulcerans* carried the *tox* gene (33/47; 70.2%). This might indicate that *C. ulcerans* acquires the toxin gene more easily or that the suspected zoonotic transmission might favor toxigenic conversion of *C. ulcerans*.

### Comparative genomics reveals zoonotic transmission of *C. ulcerans*

To address the question of whether *C. ulcerans* is a zoonotic pathogen, we analyzed nine toxigenic *C. ulcerans* isolates by NGS. The isolates form three pairs and one triplet. Within each pair we analyzed the *C. ulcerans* isolate from a human patient and one isolate from their domestic animals (for a description of the pairs see Table 1). In one case, a patient owned two cats, which were positive for *C. ulcerans*; therefore, we included an additional group, a triplet, consisting of isolates from the patient and the two cats ('pair B'). We performed resequencing with an Illumina MiSeq sequencer, and analyzed the obtained genomic information for SNPs using *C. ulcerans* 809 (GenBank CP002790) as reference genome [34]. The average coverage per genome was approximately 50-fold. Additionally, we also included other published *C. ulcerans* genomes from Brazil [34] and Japan [24] and a draft genome from France [49] for comparative genome and phylogenetic analysis.

**Table 1:** **Isolates used for sequencing in this study**

**Pair**	**Identifier**	**Host**	**Symptoms**	**G + C content**	**Assembled contigs**	**CDS**
A	KL126	Human	Throat diphtheria-like disease	53%	41	2,264
A	08-1143	Pig	Asymptomatic	53%	33	2,274
B	KL246	Human	Throat diphtheria-like disease	53%	30	2,270
B	KL251	Cat	Asymptomatic	53%	30	2,272
B	KL252	Cat	Asymptomatic	53%	34	2,270
C	KL315	Human	Ulcus, lower leg	53%	34	2,276
C	KL318	Dog	Asymptomatic	53%	31	2,269
D	KL387	Human	Wound	53%	32	2,231
D	KL392	Cat	Asymptomatic	53%	38	2,304

Isolates analyzed in this study were derived from the NCLoD at the Bavarian Health and Food Safety Authority. To our knowledge, the wounds of the patients from which the *C. ulcerans* were isolated were not caused by the animals. Additionally, given are the number of assembled contigs, their average G + C content and the number of predicted protein coding sequences (CDSs).

**Figure 2:**
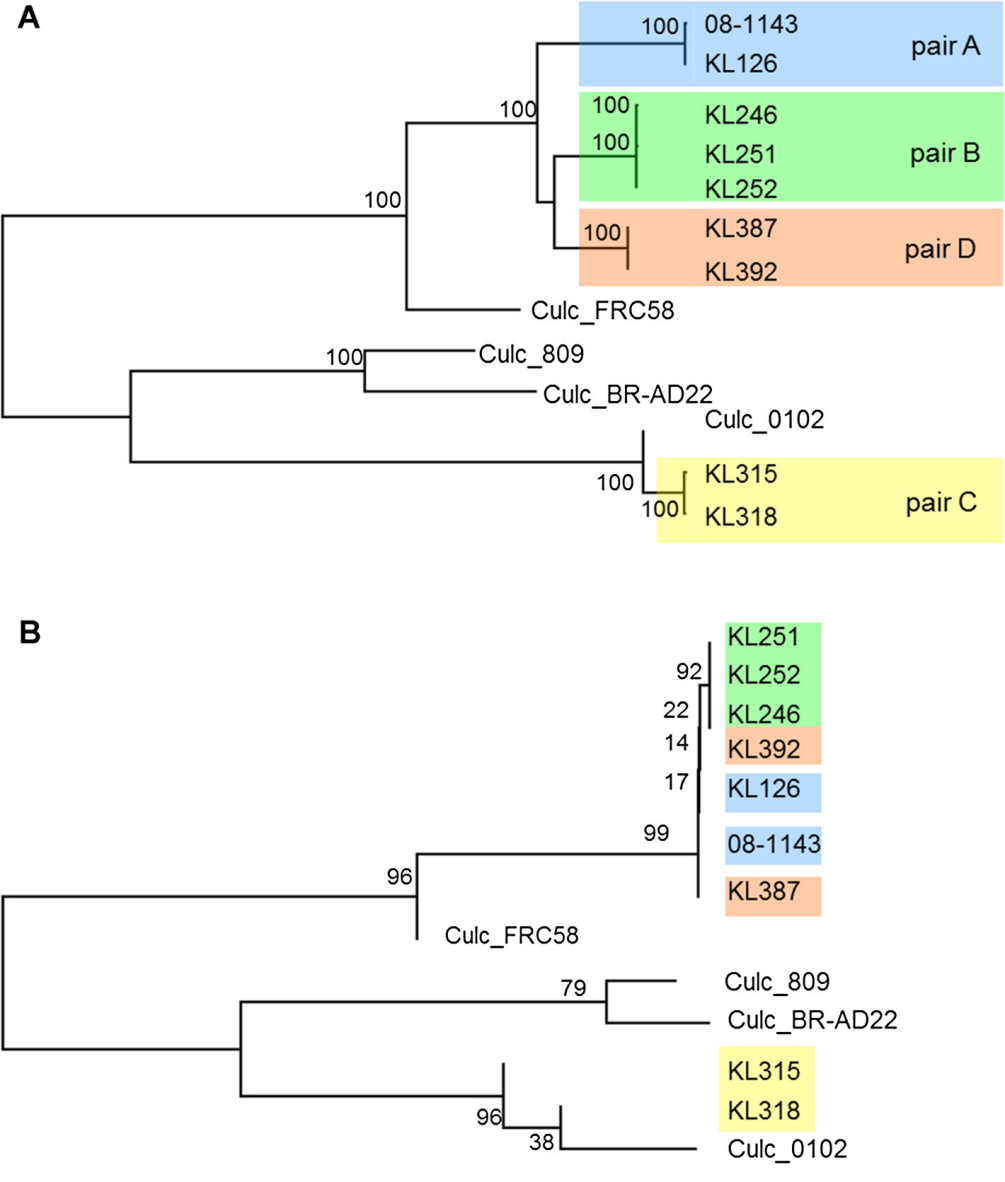
**Resequencing reveals zoonotic transmission of** ***C. ulcerans*** **and improves resolution in phylogeny compared with multi-locus sequence typing. (A)** Whole genome sequence phylogenetic analysis of the *C. ulcerans* isolates. The evolutionary history was inferred using the neighborhood joining method [55]. The percentage of replicate trees in which the associated taxa clustered together in the bootstrap test (100 replicates) are shown next to the branches [56]. The isolates within the pairs are indistinguishable from each other in the dendrogram, indicating very close relationship or even identity, while the isolates of other pairs are clearly separated **(B)** Phylogenetic analysis for seven MLST loci as in [57]. The phylogenetic analysis was conducted as in Figure 2A. KL251, KL252, KL392, KL126, 08-1143 and KL 387 fall together into one cluster which offers no information on the substructure (bootstrap values 14 to 19), showing that the resolution of MLST is not high enough to sort the isolates into the three pairs as in Figure 2A.

Interestingly, NGS revealed that *C. ulcerans* isolates from different groups varied among each other at a substantial number of SNPs (5,000 to 20,000 SNPs; Table 2) throughout the whole genome, while the isolates within a pair only showed differences at single SNPs (Table 2). SNPs found within the same group were manually curated to exclude false positive SNPs (see Methods section for details). The intra-group differences were unexpectedly small and strongly indicate that the isolates within the same group originate from a common precursor. Due to the very low number of SNPs within the groups (0 to 2 SNPs), we also conclude that zoonotic transmission took place within each group very recently (Figure 2). Interestingly, three out of four pairs from Germany and a published French draft genome of a *C. ulcerans* isolate cluster together, as also depicted by the phylogenetic analysis using the genome-wide data (Figure 2A). This result was reproducible with different phylogenetic analysis algorithms (neighbor joining, maximum parsimony, maximum likelihood; Figure S1 in Additional file 1), suggesting a European genotype for *C. ulcerans* which is different from the genotypes described from South America [34] and Asia [24]. Furthermore, we found that one pair of our collection did not cluster with the other pairs but with the genome of an isolate from Japan (Figure 2A). Remarkably in this context, our isolates clustering with the Japanese isolate (*C. ulcerans* 0102) shared one prophage with *C. ulcerans* 0102 which was shown to carry the DT encoding *tox* gene, but lacked the two other prophages identified in the *C. ulcerans* 0102 genome. Overall, we showed using NGS a zoonotic relationship in all four analyzed pairs of *C. ulcerans* isolated from humans and their domestic animals.

**Table 2:** **SNPs found in the** ***Corynebacterium ulcerans*** **isolates**

	**08-1143**	**KL126**	**KL246**	**KL251**	**KL252**	**KL315**	**KL318**	**KL387**	**KL392**	**FRC58**	**102**	**BR-AD22**
**08-1143**	0	2(35)	7,293	7,304	7,300	21,271	21,258	7,330	7,337	7,585	20,538	17,757
**KL126**	2 (35)	0	7,291	7,290	7,290	21,263	21,256	7,323	7,326	7,579	20,536	17,758
**KL246**	7,293	7,291	0	0 (46)	1 (32)	17,269	17,256	5,239	5,254	9,285	16,773	16,619
**KL251**	7,304	7,290	0 (46)	0	1 (51)	17,281	17,266	5,259	5,270	9,294	16,784	16,635
**KL252**	7,300	7,290	1 (32)	1 (51)	0	17,267	17,254	5,222	5,245	9,270	16,763	16,608
**KL315**	21,271	21,263	17,269	17,281	17,267	0	0 (96)	16,602	16,608	16,603	1,297	12,670
**KL318**	21,258	21,256	17,256	17,266	17,254	0 (96)	0	16,591	16,589	16,585	1,280	12,659
**KL387**	7,330	7,323	5,239	5,259	5,222	16,602	16,591	0	2 (37)	9,521	16,079	16,034
**KL392**	7,337	7,326	5,254	5,270	5,245	16,608	16,589	2 (37)	0	9,550	16,087	16,050
**FRC58**	7,585	7,579	9,285	9,294	9,270	16,603	16,585	9,521	9,550	0	16,316	16,166
**102**	20,538	20,536	16,773	16,784	16,763	1,297	1,280	16,079	16,087	16,316	0	12,122
**BR-AD22**	17,757	17,758	16,619	16,635	16,608	12,670	12,659	16,034	16,050	16,166	12,122	0

SNPs of the resequenced isolates show that only very minor differences are detectable within each group of isolates. Numbers in parentheses represent the number of SNPs originally given by the SNP calling pipeline. The number in front is the number of SNPs remaining after manual curation as described in the Methods section to avoid faulty SNP calling.

### Genome resequencing adds critical information to MLST

In a next step we asked whether MLST is comparable to NGS resequencing for, for example, outbreak analysis. Therefore, we compared MLST with NGS (Figure 2B): as expected by the much smaller genomic regions analyzed in MLST, we found only very few SNPs in the analyzed strains. The number of SNPs in the MLST analysis was not sufficient to discriminate pairs A and D from each other. Nonetheless, MLST recapitulated the clustering of pairs A, B and D near to the isolate from France and also found a cluster with the Japanese isolate and pair C. Noteworthy, phylogenetic analysis of the MLST data with different algorithms did not robustly reproduce the phylogenetic relationship, as indicated by low bootstrapping values (Figure 2; Figure S1 in Additional file 1). Thus, we conclude that MLST is still a helpful, fast and cost-effective tool for rough phylogenetic analysis, but NGS resequencing is superior fordetailed outbreak analysis and provides the resolution needed for in-depth understanding of transmission pathways.

### *C. ulcerans* typically carries one or more prophages

Infection of *C. diphtheriae* or *C. ulcerans* with a *tox*-carrying phage can lead to toxigenic conversion of the bacterium. Therefore, we surveyed how common prophage insertions are in *C. ulcerans* genomes. We sorted the *de novo* assembled contigs versus *C. ulcerans* 809 as reference genome and analyzed the genome for putative prophages using the PHAST algorithm [48]. We found putative prophages in most of the isolates which were sequenced in this study and also in the published *C. ulcerans* genomes (summarized in Table 3). As mentioned above, we detected the same toxigenic phage as in *C. ulcerans* 0102 in both isolates of pair C [24]. Interestingly, the other two prophages found in *C. ulcerans* 0102 were not present in pair C, isolated from a patient and a dog from Germany. In summary, we found in all isolates, except for pair A, between one and four putative prophages, suggesting that phage infection is commonly occurring in *C. ulcerans* (Table 3)*.*

**Table 3:** ***C. ulcerans*** **genome usually encode several Prophages**

**Isolate**	**Prophage identifier**	**Prophage size (kb)**	**Possible derivate of**	**Virulence factor**	**CDS**	**G + C content**	**Position (bp)**	**Att**	**Identity with other isolate of pair**	**Reference**
KL246	I	18.2	Corynephage_BFK20		18	52%	440890-459165	ND	100%	
KL251	I	18.2	Corynephage_BFK20		18	52%	441088-459363	ND	100%	
KL252	I	18.2	Corynephage_BFK20		18	52%	436472-454747	ND	100%	
KL315	I	39.3	ΦCULC0102-I	Diphtheria toxin	28	55%	142798-182119	Yes	100%	
KL318	I	39.0	ΦCULC0102-I	Diphtheria toxin	27	55%	291509-33059	Yes	100%	
KL387	I	18.2	Corynephage_BFK20	Putative RhuM	18	52%	438665-456940	ND	ND	
	II	39.8	Rhodococcus phage REQ2		50	54%	1898856-1938678	Yes	100%	
	III	10.4	Mycobacterium phage Fishburne		17	52%	2527900-2538480	ND	92%	
KL392	I	42.1	Rhodococcus phage REQ2		54	55%	1858755-1900873	Yes	100%	
	II	10.4	Mycobacterium phage Fishburne		17	52%	2505077-2515476	ND	92%	
102	I	38.3	ΦCULC0102-I	Diphtheria toxin	26	54%	168523-206883	Yes		[24]
	II	21.4	ΦCULC0102-II		18	52%	536771-558192	ND		[24]
	III	39.4	ΦCULC0102-III		22	57%	1377963-1417370	Yes		[24]
BR-AD22	I	42.0	ΦCULC22-I		42	53%	1299138-1338708	ND		[34]
	II	44.9	ΦCULC22-II		60	55%	1853009-1877311	Yes		[34]
	III	14.0	ΦCULC22-III		19	57%	1963728-1986514	Yes		[34]
	IV	41.0	ΦCULC22-IV		53	54%	2134999-2156991	Yes		[34]
809	I	41.4	ΦCULC809-I		45	53%	1295507-1335046	ND		[34]
FRC58	I	29.4	Mycobacterium phage Fishburne		52	53%	2493492-2522907	Yes		[49]

Prophages are as annotated by PHAST [48] or in [24,34]. Given are the isolate and prophage identifiers, the predicted prophage size and the name of the prophage sharing the highest similarity with the predicted prophage. The shared identity of the prophage with the corresponding pair isolate is given as a percentage. The prophage KL387-III was not predicted by PHAST, most likely due to a contig boundary. However, the alignment with KL392-III clearly identified the prophage region KL387-III with 100% identity. KL387-I was blasted versus the whole genome data of KL392 but no similarity was identified, arguing against a false negative detection of this prophage in the KL392 genome. Att.: predicted attachment site of the phage. CDS, coding sequence.

**Figure 3:**
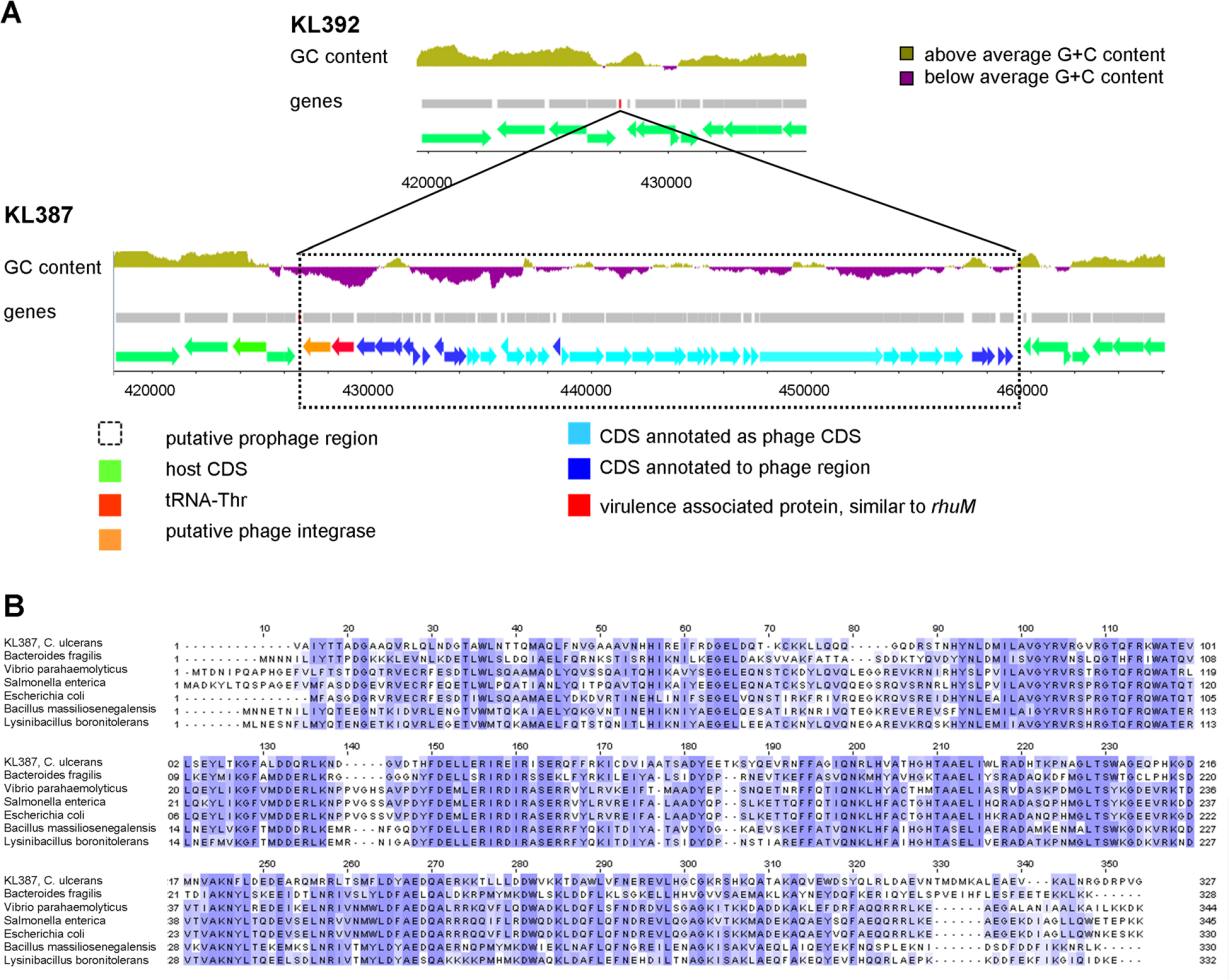
**Phage infection of** ***C. ulcerans*** **can rapidly change its pathogenicity. (A)** Genome Browser view of the annotated prophage region of KL387 and the corresponding region in KL392. The tRNA-Thr locus, which most likely serves as an integration site, is shown in red in the upper panel. The upper lane in both panels reflects the local GC content. In the region of the prophage the GC content is below the average GC content of *C. ulcerans*, as indicated by the purple color. Predicted genes are depicted as arrows below the GC content. Among other known prophage proteins we identified a phage integrase and a potential virulence factor sharing high identity with RhuM (45%) in the prophage of KL387. The dashed box indicates the putative prophage locus. **(B)** The additional prophage of KL387 contains a putative virulence factor similar to RhuM of *Salmonella enterica*. Multiple alignments of the putative virulence factor from KL387 (first row) with the RhuM virulence factor from *Bacteroides fragilis* (EXY75214.1), *Vibrio parahaemolyticus* (EVT77386.1), *S. enterica* (ESE75243.1), and *Escherichia coli* (EZJ48339.1) and the Fic toxin from *Bacillus massiliosenegalensis* (WP_019154237.1) and *Lysinibacillus boronitolerans* (WP_016992295.1). The amino acid sequences have been colored according to their similarity score according to the blosom 62 matrix: dark blue reflects identity, light blue a positive score and white no identity. CDS, coding sequence.

In a next step we compared the putative phage content of the individual isolates forming a human-animal pair and found that the predicted prophage content was nearly identical. We found only that KL387 and KL392 (pair D) differ in their putative prophage content (Figure 3A), although the SNP analysis of the human-animal isolate pair showed only very minor differences (two verified SNPs in approximately 2.5 Mb). This finding strongly indicates that both isolates originate from the same parental *C. ulcerans* strain and the very low number of detected SNPs argues for a recent event of phage integration, likely because there was insufficient time to acquire new SNPs in the meantime. The additional putative prophage in KL387 is integrated just downstream of the tRNA-Thr locus (anticodon: CGT) and is flanked by an 85 bp direct repeat with 100% identity (426.686-426.771 and 459378-459463 bp in KL387). One of the two repeats is, as expected, also present in KL392. The integration near a tRNA locus and the duplication of a short genomic region flanking the integration region of the prophage are typical features found at prophage integration sites in many bacteria [58]. Additionally, the local GC content in the putative prophage region of KL387 is considerably lower than the GC content of the genomic region surrounding the putative prophage. This is typically found at prophage integration sites [58] and strongly suggests an event of horizontal gene transfer in this region.

Furthermore, closer analysis of the predicted genes in the putative prophage revealed, for all predicted sequences, known phage homologues or proteins associated with putative prophages from other bacteria (Figure 3B). Excitingly, we found one predicted protein that shows high identity to the Fic toxin of *Bacillus massiliosenegalensis* and to the RhuM virulence factor from the *Salmonella enterica* pathogenicity island 3 (SPI-3). RhuM (NP_462654) and the predicted phage protein shared 42.3% identity and 58.3% similarity (Figure 3C). It was shown that RhuM inactivation leads to highly reduced virulence of *Salmonella* and to a lower mortality rate after *S. enterica* infection in the *Caenorhabditis elegans* model [59]; however, no clear molecular function for this protein is known. Therefore, increased virulence of KL387 versus KL392 caused by the integration of the phage remains to be shown. We hypothesize, however, that the conversion by a virulence factor- or toxin-carrying phage of *C. ulcerans* can take place very rapidly and might change the virulence of the strain even within short periods of times - for example, even within a single zoonotic transmission event.

### A novel, putative diphtheria toxin-encoding pathogenicity island in *C. ulcerans*

In the isolates KL315 and KL318 (forming pair C) the DT-encoding *tox* genes were located in a predicted prophage region which exhibits very high identity to the toxigenic prophage of *C. ulcerans* 0102 (99% identity) [24]. Conversion of a non-toxigenic to a toxigenic bacterium by prophage integration is well described for *C. diphtheriae* and is also assumed to take place in *C. ulcerans.*

**Figure 4:**
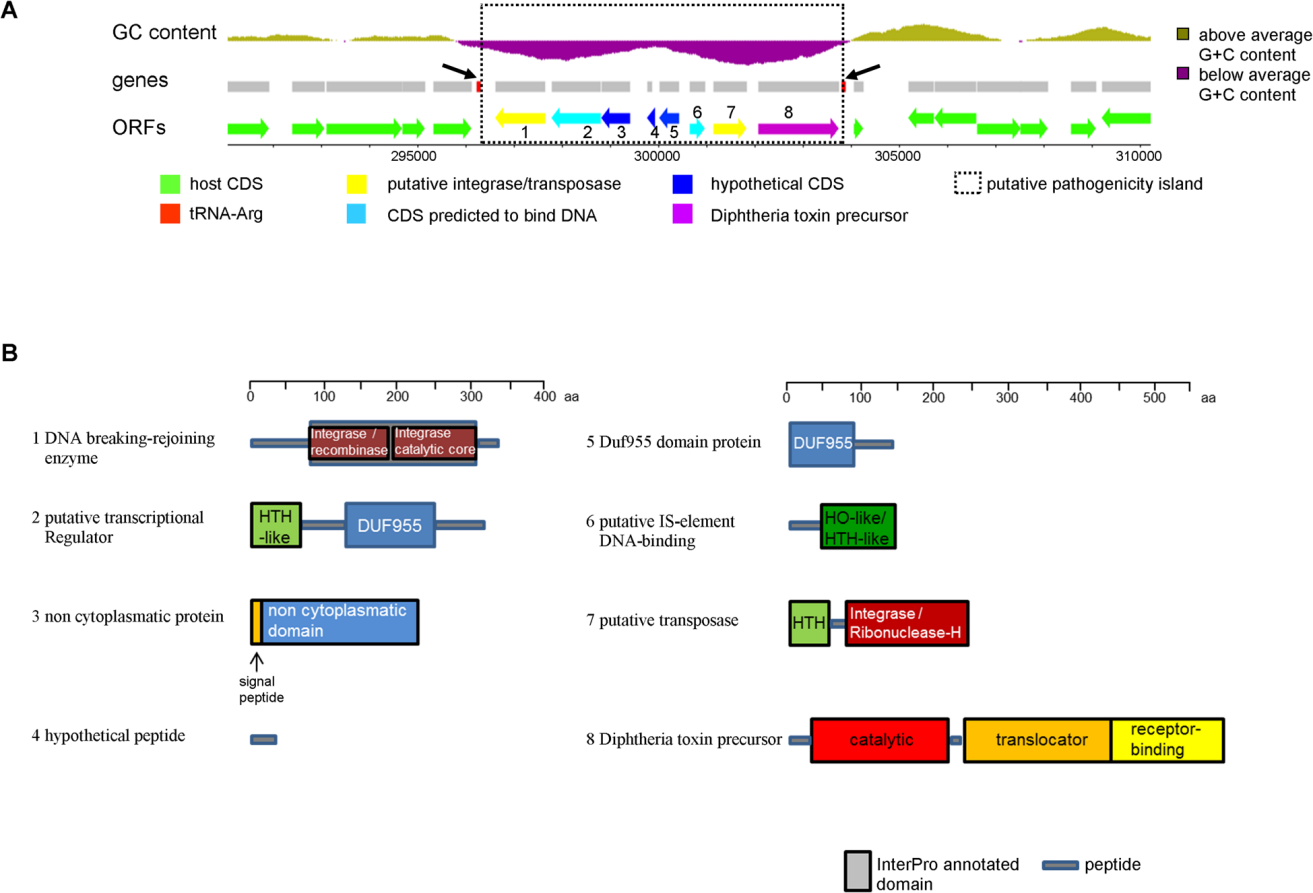
**A novel pathogenicity island encoding the diphtheria toxin in** ***C. ulcerans*** **. (A)** Genome Browser view of the novel PAI of KL251. The upper panel indicates the lower local GC content of the PAI compared with the remaining *C. ulcerans* genome. The borders of the lower GC regions delimit the novel genomic region. The regions up- and downstream of the PAI are conserved in other *Corynebacterium* species and are flanked by a direct and near perfect 100 bp repeat, which includes parts of the tRNA-Arg and thereby results in a pseudo-tRNA gene downstream of the PAI. Both tRNA-Arg and the pseudo-tRNA are labeled with black arrows. Among the predicted proteins of the PAI are two putative integrases/transposases and two additional predicted DNA binding proteins and the DT. CDS, coding sequence. **(B)** Predicted domains of the proteins. The locations of the proteins in the PAI are indicated by the numbers in (A). (1) A predicted integrase/recombinase enzyme. (2) A putative transcriptional regulator carrying a DUF955 domain with unknown function. The DUF955 domain carries a H-E-X-X-H motif and is suspected to be catalytically active as metallohydrolase [64]. The helix-turn-helix (HTH)-like domain is similar to the HTH-like domain of the Cro/C1 and lambda repressor. (3) A non-cytoplasmic protein of unknown function with predicted signal peptide. (4) Hypothetical peptide, which is most likely not expressed. (5) Protein with a DUF955 domain of unknown function. (6) Possible homologous protein to a putative insertion element (IS): homeodomain (HO)-like domain including a HTH-domain. Predicted to bind a specific DNA sequence and suspected to be a transcriptional regulator [65]. (7) Putative transposases composed of a DNA-binding HTH domain and an integrases/ribonuclease H domain. (8) DT precursor as known from other *C. ulcerans* and *C. diphtheriae* isolates.

Additionally, we found in seven out of nine toxigenic isolates a novel, unknown and putative PAI harboring the DT encoding gene (Figure 4A): the novel, putative PAI was present in KL126, 08-1143, KL246, KL251, KL252, KL387, and KL392 and is in all seven strains located at the same genomic site, just downstream of the tRNA-Arg (anticodon: ACG). Interestingly, this locus is known to be targeted by many events of horizontal gene transfer: the toxigenic prophages from *C. ulcerans* 0102 [24], KL315 and KL318 are integrated into this locus. Additionally, a putative virulence factor has been found at this genomic position in *C. ulcerans* 809 and was hypothesized to be a ribosome binding protein that shares high similarity with the Shiga toxin [34], which we were unable to detect it in our isolates. Furthermore, this conserved tRNA site is described in *C. diphtheriae* as an integration site for toxigenic and other prophages [60-62] and it seems that this integration hot spot in the *Corynebacterium* genome is highly conserved, as it has been reported that phage integration can take place at this tRNA locus in at least three different *Corynebacterium* species [63].

We initially identified the novel, putative toxigenic PAI by analysis of the local GC content, which is strongly reduced in a region around the DT gene. The putative PAI localizes just downstream of a tRNA-Arg (anticodon: ACG) and parts of the tRNA have been duplicated leading to a predicted pseudo-tRNA at the 3′ end of the PAI, with a perfect 100 bp directed repeat. Comparison with other available genome data and analysis of the duplicated region within the putative PAI suggest a size of 7,571 bp for the PAI. The GC content of approximately 48% compared with an average GC content of approximately 53% for the whole genome of *C. ulcerans* together with the 100 bp directed repeat strongly indicate horizontal gene transfer [66]. The novel *C. ulcerans* PAI was predicted to contain eight proteins. Most interestingly, among these we found the DT precursor (Figure 4B). It is located to the 3′ end of the PAI just upstream of the pseudo-tRNA. The *tox* gene is >99% identical to the alleles described for *C. ulcerans* [67]*.* We found for several of the isolates (for example, KL126 and 252) that the DT was expressed in sufficient amounts to result in positive signals in the Elek test, indicating functional DT expression. Additionally, a protein of the PAI was predicted to be a transposase and the adjacent gene was predicted to encode a protein containing a homeodomain-like (HO-like) domain with a helix-turn-helix (HTH)-like motif. This protein shares high similarity with known insertion elements from other *Corynebacterium* species. Bioinformatics analyses suggest that it might serve as a transcriptional regulator by sequence-specific DNA binding via its HO-like domain (Figure 4B). Furthermore, we identified a putative integrase/Tyr-recombinase and a putative transcription regulator containing an HTH-like domain (Figure 4B). HTH motifs are known to bind DNA in a sequence-specific manner. In addition to the HTH-like domain, this protein also carries a DUF955 domain which has no known function but is suspected to be catalytically active, since the H-E-X-X-H motif might bind metal ions and serve as a hydrolase (Figure 4B). Remarkably, among the eight predicted polypeptides of this novel, putative PAI we found a second putative protein of unknown function carrying a similar DUF955 domain (Figure 4B). This novel, putative PAI is highly conserved within the seven isolates. We only detected one SNP within this PAI within all seven isolates, showing its high conservation.

## Discussion

The presented study of nine *C. ulcerans* draft genomes demonstrates for the first time the zoonotic transmission of toxigenic *C. ulcerans* on a molecular level, which was previously predicted by sequence data of single gene fragments and ribotyping. We report that pairs of patient and companion/domestic animal isolates of *C. ulcerans* show no or only very few differences in their genome-wide SNP profiles, while the isolates obtained from different patients and/or animals show many more differences. This proves that *C. ulcerans* undergoes zoonotic transmission between animals and humans. Additionally, the results illustrate that analysis by NGS improves the toolkit for phylogenetic and epidemiological studies, by adding more detailed information, more resolution and more robust discrimination between closely related isolates.

Moreover, our data show that *C. ulcerans* isolates often carry one or more prophages which are able to modify pathogenicity of the bacteria. Interestingly, we found that even within the pair of isolates derived from a patient (KL387) and their cat (KL392), phage integration can take place. Even though both isolates do not differ from each other in their SNP profiles (we only detected two SNPs) and indels, we found that the isolate from the human patient carried a prophage. Since we could not detect any remnants or duplicated sequences in KL392 in proximity to the tRNA-Thr locus, where the prophage is integrated in KL387, we suppose that the prophage was integrated into KL387 rather than excised from KL392. In addition, we found a putative virulence factor among the predicted proteins of the prophage. This protein shared high identity with RhuM, a protein from *S. enterica*. It was shown in a *C. elegans* model to be important for epithelial cell invasion of *S. enterica* [59]. A molecular function for RhuM in *S. enterica* is not known, but sequence analysis points towards DNA-binding activity [68]. Additionally, deletion of *rhuM* reduced the fraction of killed *C. elegans* upon *Salmonella* infection by approximately half [59]. We did not assay for changed pathogenicity of the isolates carrying the *rhuM* homologous gene but it would be very interesting to know whether *rhuM* expression leads also to higher virulence of *C. ulcerans* similar to *S. enterica*, using an *C. ulcerans* infection model [69]. Nevertheless, here we provide evidence that prophages can be taken up and integrated very rapidly into the *C. ulcerans* genome, in the reported case even within one zoonotic transmission event. As a consequence, this might lead to a change in virulence and pathogenicity of *C. ulcerans*. We showed that NGS analysis is able to identify such novel gene acquisitions and other genomic modifications in bacteria very efficiently. This strongly underlines that, for detailed and comprehensive epidemiologic surveillance and monitoring of pathogens, NGS analysis represents a very effective tool to identify emerging critical changes in the virulence of bacteria.

Furthermore, considering the higher proportion of toxigenic versus non-toxigenic *C. ulcerans* compared with *C. diphtheriae*, we found that seven out of nine analyzed *C. ulcerans* isolates carried a putative PAI which is completely different to the known prophages encoding DT. To our knowledge no case of a *Corynebacterium* carrying a DT gene which is not located in a prophage region has been described to date. There are indications that the putative PAI might be inserted by horizontal gene transfer into a recombination hot spot in the *Corynebacterium* genome. This recombination hotspot has been described for several *Corynebacterium* species [63]. Firstly, we found that the GC content of the PAI region differed from the remaining genome. Secondly, we found putative integrases/recombinases and terminal direct repeats (Figure 4A), duplicating parts of the tRNA-Arg adjacent to the putative PAI. Since this genomic site is highly conserved in several *Corynebacterium* species and is known to serve for other integration events as a target/attachment site (for example, for prophages), it would be interesting to analyze other toxigenic *Corynebacterium* species to see if they also contain this novel, putative PAI or a similar insert. Alternatively, this PAI could be specific to *C. ulcerans* and might, therefore, be the reason for the higher proportion of toxigenic *C. ulcerans.*

The finding of the novel *tox* gene encoding a putative PAI leads to the very important question for future research of whether the whole identified PAI forms a functional unit. One hypothesis is that the PAI is a large 'hybrid transposon', encoding a transposase and other recombination enzymes, which targets the tRNA-Arg recombination site. Containing the DT gene, it may represent an additional virulence factor which can spread by horizontal gene transfer. Another possibility would be that the PAI originated by several events. For instance, it can be speculated that several insertion elements, one of which carried the *tox* gene, integrated into this genomic site. However, since we found seven identical PAIs in nine toxigenic isolates, which differed to a larger extent in the remaining genome, we favor the hypothesis that the putative PAI itself might be a genomic element which can be transferred horizontally between *C. ulcerans*. If the PAI developed in several strains in parallel, we would expect less conservation and more SNPs and most likely different compositions for it between the different pairs of isolates. The idea of horizontal transfer is supported by the finding that the PAI contains genes for two integrase/transposase-like proteins and at least two additional predicted DNA-binding proteins, which share similarity with proteins involved in horizontal gene transfer (phages/insertion elements). Such proteins would be expected in a putative 'hybrid transposon' which could insert to a target site via the site-specific binding/action of its encoded proteins. An efficient horizontal transfer mechanism could also well explain why such a large fraction of the isolates are toxigenic and the high conservation of the novel PAI.

Furthermore, it is an interesting point to speculate why the proportion of toxigenic and non-toxigenic strains among *C. ulcerans* outnumbers that of C*. diphtheriae* in our strain collection. A possible hypothesis is that this PAI is specific for *C. ulcerans* and that it spreads much more efficiently than the toxigenic phage. Additional factors influencing the proportion of toxigenic/non-toxigenic bacteria might be zoonotic maintenance, which might favor the emergence of toxigenic species by an unknown mechanism or the more moderate toxin expression in *C. ulcerans* which might be favorable for better host adaption than higher toxin levels such as produced by *C. diphtheriae*.

## Conclusions

We prove the hypothesis that *C. ulcerans* is transmitted by a zoonotic pathway based on molecular data using a whole genome sequencing approach. To better understand the virulence potential of *C. ulcerans*, we inspected genome sequencing data for possible events of horizontal gene transfer which could cause increased virulence of *C. ulcerans* strains. We show that acquisition of virulence factors can take place very rapidly, as demonstrated by a phage integration event carrying a putative virulence factor, similar to a virulence factor known from *S. enterica*. This finding illustrates the importance of methods such as NGS in epidemiology, which can detect novel gene acquisitions, which can have a high impact on the virulence of pathogens. Additionally, we identified a novel, putative PAI which could potentially be subjected to horizontal gene transfer and thereby explain the high fraction of toxigenic *C. ulcerans*. This PAI is, to our knowledge, the first example of a DT gene locus not associated with a prophage and will be very important for understanding the pathogenesis of diphtheria-like disease caused by *C. ulcerans*. For the future it will be crucial to analyze this novel, putative DT transmission pathway in molecular detail to understand the emerging pathogen *C. ulcerans.*

## Additional file

Additional file 1: Figure S1. Phylogenetic analysis of whole genome sequencing (WGS) and MLST data with additional methods: maximum likelihood, maximum parsimony and neighborhood joining algorithms.

## Abbreviations

DT: diphtheria toxin
HO: homeodomain
HTH: helix-turn-helix
MLST: multi-locus sequence typing
NCLoD: National Consiliary Laboratory on Diphtheria
NGS: next generation sequencing
PCR: polymerase chain reaction
PAI: pathogenicity island
SNP: single nucleotide polymorphism
