# Supplementary material for: Next generation sequencing analysis of nine Corynebacterium ulcerans isolates reveals zoonotic transmission and a novel putative diphtheria toxin-encoding pathogenicity island
Source: Genome Med. 2014 Nov 28;6(11):113. doi: 10.1186/s13073-014-0113-3 (PMC4293111; doi:10.1186/s13073-014-0113-3)
Supplement: Additional file 1: Figure S1. — Phylogenetic analysis of whole genome sequencing (WGS) and MLST data with additional methods: maximum likelihood, maximum parsimony and neighborhood joining algorithms. [file 13073_2014_113_MOESM1_ESM.pdf]

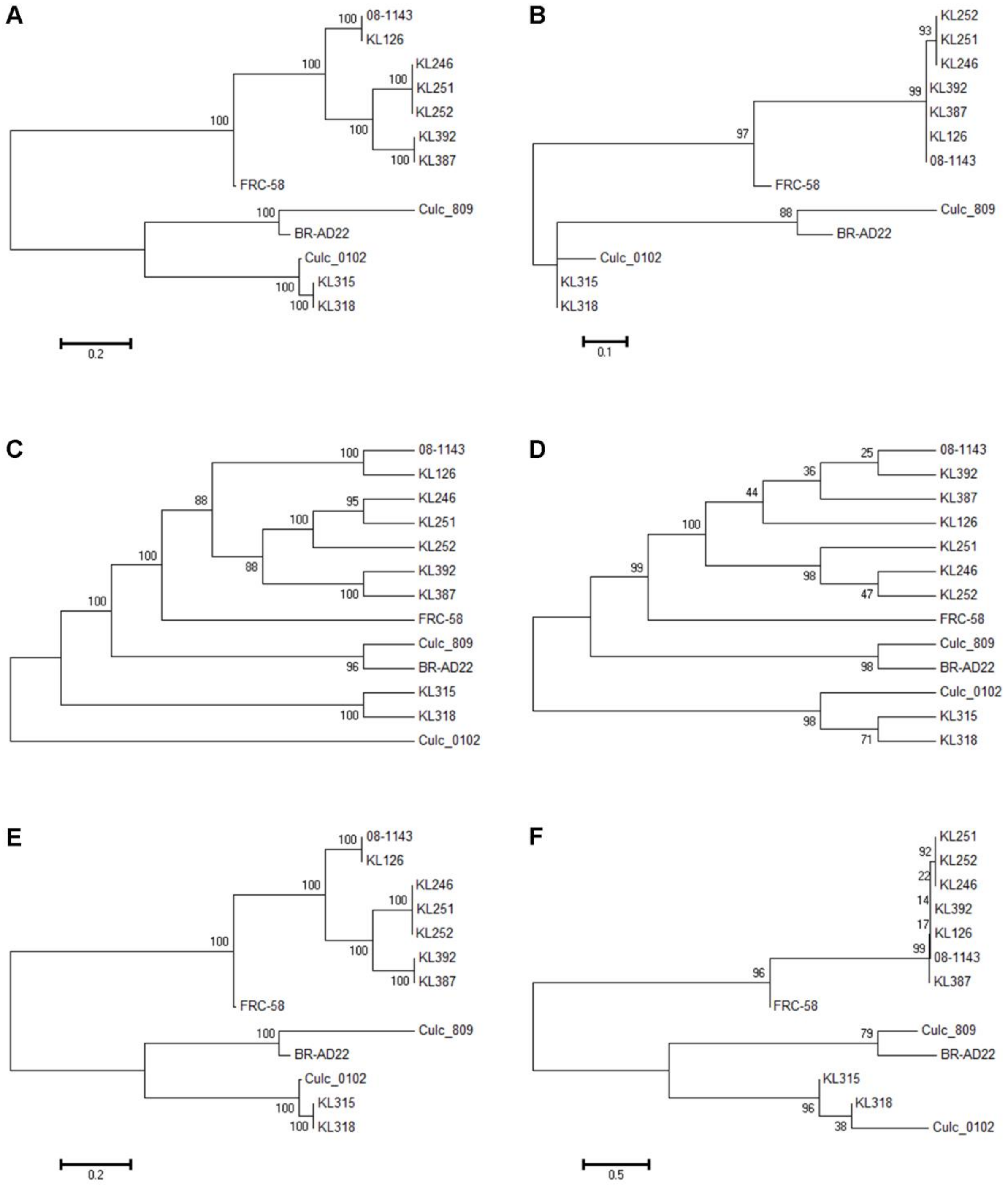

Additional Figure 1: Phylogenetic analysis of whole genome sequencing (WGS) and MLST data

Phylogenetic analysis of the *C. ulcerans* isolates. The evolutionary history was inferred using different algorithms with MEGA 6.0[1]. The percentage of replicate trees in which

the associated taxa clustered together in the bootstrap test (100 replicates) are shown next to the branches [2]. Shown are on the left panel trees which are based on the SNPs of the whole genome sequencing data (WGS), on the right side the data for the 7 MLST loci, similar to **Figure 2**. **(A)** Maximum likelihood phylogeny based on WGS data **(B)** Maximum likelihood phylogeny based on MLST data **(C)** Maximum Parsimony phylogeny based on WGS data **(D)** Maximum Parsimony phylogeny based on MLST data **(E)** Neighbor joining phylogeny based on WGS data **(F)** Neighbor joining phylogeny based on MLST data. The dendrograms for the WGS illustrate the higher resolution of WGS versus MLST data and also the higher accuracy and robustness as shown by much higher bootstrap values especially at the cluster of 08-1143, KL126, KL246, KL251, KL 252, KL387 and KL392. Note that MLST based methods completely fail to identify the matched pairs within this cluster and that the extremely low bootstrapping values indicate uncertainty in this cluster for MLST while WGS data provides almost perfect bootstrapping values.

1. Tamura K, Stecher G, Peterson D, Filipski A, Kumar S (2013) MEGA6: Molecular Evolutionary Genetics Analysis version 6.0. Mol Biol Evol 30: 2725-2729.
2. Efron B, Halloran E, Holmes S (1996) Bootstrap confidence levels for phylogenetic trees. Proc Natl Acad Sci U S A 93: 7085-7090.
